# Assessment of Carrier-Free Metallacarboranes for Targeted Radiation Therapies PBFT and BNCT: Comparative Cellular Effects and Dosimetry Studies with [*o*-FESAN]^−^ in Breast Cancer Cells

**DOI:** 10.3390/ph18101491

**Published:** 2025-10-03

**Authors:** Salvatore Di Maria, Teresa Pinheiro, Luís Cerqueira Alves, Valeria Bitonto, Nicoletta Protti, Simonetta Geninatti Crich, Kai Nishimura, Hiroyuki Nakamura, António P. Matos, Catarina I. G. Pinto, Filipa Mendes, Francesc Teixidor, Clara Viñas, Fernanda Marques

**Affiliations:** 1 Centro de Ciências e Tecnologias Nucleares (C2TN), Instituto Superior Técnico, Universidade de Lisboa, Estrada Nacional 10, 2695-066 Bobadela, Portugal; lcalves@ctn.tecnico.ulisboa.pt (L.C.A.); catarina.pinto@tecnico.ulisboa.pt (C.I.G.P.); fmendes@ctn.tecnico.ulisboa.pt (F.M.); 2 Departamento de Engenharia e Ciências Nucleares (DECN), Instituto Superior Técnico, Universidade de Lisboa, Estrada Nacional 10, 2695-066 Bobadela, Portugal; murmur@ctn.tecnico.ulisboa.pt; 3 Instituto de Bioengenharia e Biociências (iBB), Instituto Superior Técnico, Universidade de Lisboa, Av. Rovisco Pais 1, 1049-001 Lisboa, Portugal; 4 Dipartimento di Biotecnologie Molecolari e Scienze per la Salute, Centro Imaging Molecolare, via Nizza 52, 10126 Torino, Italy; valeria.bitonto@unito.it (V.B.); nicoletta.protti@unipv.it (N.P.); simonetta.geninatti@unito.it (S.G.C.); 5 School of Life Science and Engineering, Institute of Science Tokyo, 4259 Nagatsuta-cho, Midori-ku, Yokohama 226-8501, Japan; nishimura.k.am@rakumail.jp; 6 Laboratory for Chemistry and Life Science, Institute of Integrated Research, Institute of Science Tokyo, 4259 Nagatsuta-cho, Midori-ku, Yokohama 226-8501, Japan; hiro@cls.iir.isct.ac.jp; 7 Centro de Investigação Interdisciplinar Egas Moniz, Campus Universitário, Quinta da Granja, Monte de Caparica, 2829-511 Caparica, Portugal; apamatos@gmail.com; 8 Institut de Ciència de Materials de Barcelona (CSIC), Campus Universitat Autònomade Barcelona (UAB), 08193 Bellaterra, Barcelona, Spain; teixidor@icmab.es (F.T.); clara@icmab.es (C.V.)

**Keywords:** Ferrabis(dicarbollide), cancer treatment, particle radiation therapies, proton boron fusion therapy (PBFT), boron neutron capture therapy (BNCT), breast cancer

## Abstract

**Background:** Ferrabis(dicarbollide) ([*o*-FESAN]^−^) in combination with proton–boron fusion therapy (PBFT) or boron neutron capture therapy (BNCT) are promising alternative radiation modalities for the treatment of breast cancer. The aim of this study was to explore the underlying effects of [*o*-FESAN]^−^ radio enhancement on breast cancer cells in vitro and in vivo, and to perform comparative dosimetry calculations. **Methods:** The cellular effects on SKBR-3 and MDA-MB-231 breast cancer cells and MDA-MB-231 xenograft-bearing nude mice induced by carrier-free [*o*-FESAN]^−^ after BNCT or PBFT were evaluated following recommended protocols. Monte Carlo (MC) dosimetry calculations were performed at the cellular scale for both radiation modalities. **Results:** Selective retention of [*o*-FESAN]^−^ within the cytoplasm and nucleus of SKBR-3 and MDA-MB-231 breast cancer cells is demonstrated. Moreover, in vivo studies with MDA-MB-231 xenograft-bearing nude mice show appreciable accumulation of [*o*-FESAN]^−^ in the tumor. Both radiation modalities induce loss of cellular viability and survival. Comparative dosimetry studies between proton and neutron irradiation agree with the viability data, showing a good correlation between absorbed dose vs. cellular effects. In the case of PBFT, cell structural changes are likely due to necrosis caused by the production of reactive oxygen species (ROS). To explain the radio enhancement effects in more detail, other mechanisms should be taken into consideration. **Conclusions:** Our results validate the effectiveness of both PBFT and BNCT therapeutic modalities, warranting further studies on carrier-free [*o*-FESAN]^−^ as a candidate drug for potential clinical translation of radio enhancers in binary radiation therapies.

## 1. Introduction

**Scheme 1:**
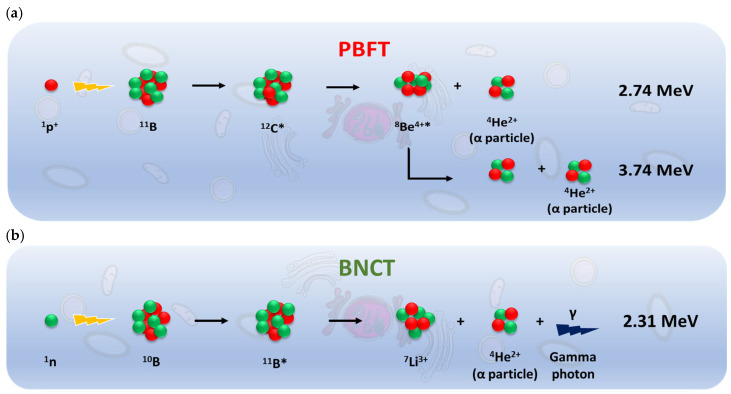
(**a**) PBFT—the reaction between an energetic proton and ^11^B results in the formation of an excited ^12^C nucleus (^12^C*), which then splits into a 2.74 MeV alpha (α) particle and an unstable ^8^Be (^8^Be^4+^*) nucleus, which rapidly decays into two alpha particles [25]; (**b**) BNCT—the reaction between a neutron and ^10^B results in the formation of an excited ^11^B^*^ nucleus, which then splits into a ^7^Li nucleus and a 2.31 MeV alpha (α) particle.

Particle therapy (PT) is a new weapon in radiation therapy (RT), especially to treat tumors that are radioresistant to conventional photon beam radiotherapy [1,2,3]. Due to its non-invasive nature (mainly due to the Bragg peak characteristics), PT is able to deliver high radiation doses to the tumor, while reducing exposure to healthy tissue. Proton–boron fusion therapy (PBFT) and boron neutron capture therapy (BNCT) are particle therapies currently under experimental evaluation or experimental cancer treatment, and have been reported to improve therapeutic outcome, while minimizing radiation-related toxicities [4,5,6]. PBFT is based on the nuclear fusion between ^11^B nuclei and incident beam protons (Scheme 1a) [4]. The reaction is characterized by a significant resonance near the Bragg peak at approximately 0.675 MeV with a cross section of about 1 barn. The emission of three high-LET α-particles that occur primarily in the Bragg peak region allows for deposition of maximum energy at the tumor site, while minimizing the damage to normal cells and tissues along their path. The direct-killing effect of ions and irreparable DNA damage permits the eradication of cancer cells even in hypoxic tumors [7,8,9,10,11,12,13].

BNCT is based on the nuclear capture and fission reaction that occurs when a stable boron isotope (^10^B) is irradiated with thermal neutrons (<0.5 eV) to yield high-LET α particles (^4^He) and recoiling ^7^Li nuclei (Scheme 1b) [14,15,16,17]. A sufficient amount of ^10^B must be delivered to the tumor (~10^9^ atoms/cell; ~20–30 µg/g tumor) to generate a lethal effect upon neutron irradiation. The B-N reaction takes place following two channel reactions. In the first channel (6%), the energies of the Li and α-particles produced are 1.01 MeV and 1.78 MeV, respectively. In the second channel reaction (94%), the energy of the alpha particle produced is 1.47 MeV, while the energy of the Li-particle is 0.84 MeV. The remaining energy, 0.48 MeV, is gamma-ray energy [18].

Despite the potential therapeutic advantages, challenges associated with the general use of BNCT include the limited availability of suitable neutron beams, lack of selective boron compounds, and limited knowledge regarding radiation dosimetry and radiobiological effects [19,20,21,22].

In principle, PBFT has advantages over both BNCT and proton therapy (PT) and could represent a more effective treatment modality [5]. Using the boron drugs originally developed for BNCT, experimental reports showed a significant increase in cell-killing effectiveness of proton beam irradiation of ^11^B-containing cancer cells [11,12,13,23]. However, the reported evidence for the possibility of using boron compounds for dose enhancement in PT, as well as their radiosensitizing effect, is still in its infancy towards clinical applications [4,5]. In fact, the uncertainty regarding the dose distribution and the relative biological effectiveness (RBE) of protons, which is assumed to be 1.1 versus that of photons (1.0), questions proposed mechanisms underlying PBFT effectiveness [7,8,9,10,11,12,24]. In addition, in vitro studies with cellular models showed that the radiation-induced effects vary with the type of cell, which might explain the inconclusive data reported in the literature. Although the concept of enhancing the radiobiological effects of proton therapy, while keeping its unique ballistic properties, is quite appealing and of potential clinical relevance, it clearly needs further research [4].

Boron carriers have emerged as one of the most promising agents for BNCT as a source of sensitizers to achieve local and enhanced therapeutic effects. Although a considerable number of multifunctional boron agents have been investigated, only a few have been advanced to clinical studies [25,26,27,28,29,30,31,32,33]. Currently, only two boron compounds, ^10^B-BPA (Steboronine^®)^) (Stella Pharma, Singapore, Vietnam) and ^10^B-BSH (sodium borocaptate), are being used in clinical trials. Nevertheless, neither one of them adequately fulfills the criteria of an ideal boron delivery agent, i.e., BPA suffers from low boron content and BSH lacks tumor selectivity [32].

**Chart 1:**
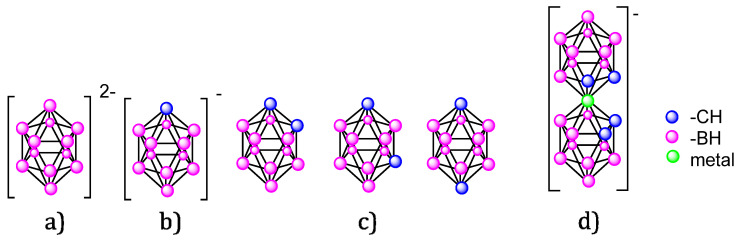
Schematic representation of icosahedral boranes and heteroborane clusters. (**a**) Icosahedral *closo* dianionic boranes ([B_12_H_12_]^2−^); (**b**) monoanionic *closo* carboranes ([1-CB_11_H_12_]^−^); (**c**) neutral *closo* dicarboranes (1,2-C_2_B_10_H_12_, 1,7-C_2_B_10_H_12_ and 1,12-C_2_B_10_H_12_); (**d**) monoanionic metallabis (dicarbollides) M = Co^3+^, Fe^3+^ [3,3′-M(1,2-C_2_B_9_H_11_)_2_]^−^ in its *cisoid* rotamer.

The amount of boron is crucial to fulfill the requirement of an adequate ^10^B in the tumor cell to enhance BNCT efficacy [32]. Towards this goal, icosahedron clusters have been used in the development of a new generation of BNCT drugs (Chart 1) [34,35,36,37,38,39,40,41,42,43,44,45,46,47,48,49,50,51,52]. Some of these boron-rich structures include cellular and DNA targeting molecules [27], macro/nano-vehicles and boron carriers [28,29,30,31], boron immunoadjuvants [48,49], and imaging radionuclides for theranostic applications [34,52].

Metallabis(dicarbollides), [3,3′-M(1,2-C_2_B_9_H_11_)_2_]^−^ (M = Co, Fe), abbreviated as [*o*-COSAN]^−^ or [*o*-FESAN]^−^, have adequate physicochemical properties that, combined with their boron-rich content, make them promising compounds as a boron source for BNCT [43] and PBFT [25]. Their good solubility in water (approx. 1 M for Na[*o*-FESAN]) [53,54], biocompatibility of the counterion (Na), biological stability [55], and the capacity to interact with DNA [42,56] meet the requirements to explore pre-clinical evaluation.

**Chart 2:**
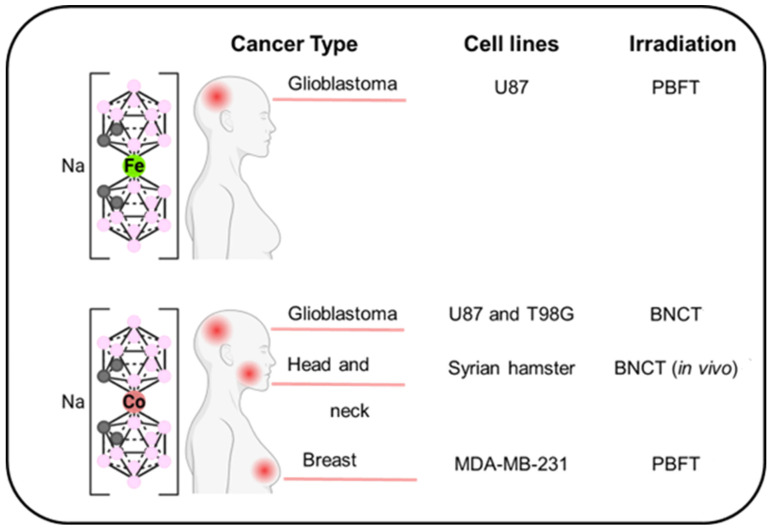
Previous work supporting the potential use of metallabis(dicarbollides) ([*o*-COSAN]^−^ or [*o*-FESAN]^−^) in prospective BNCT or PBFT applications [25,43,44,57].

Previous work has demonstrated the potential of [*o*-COSAN]^−^ or [*o*-FESAN]^−^ as multifunctional chemotherapeutics with prospective application in BNCT or PBFT (Chart 2) [25,43,44,57]. The cell-killing effects induced by PBFT with [*o*-FESAN]^−^ in glioblastoma cells suggest PBFT as a promising radiation modality to fight tumors that are difficult to treat [25].

In this work, we performed in vitro studies with SKBR-3 and MDA-MB-231 breast cancer cells, following recommended evaluation methods, and evaluated the cellular effects induced by [*o*-FESAN]^−^ after BNCT or PBFT [36,37]. Furthermore, biodistribution studies in a MDA-MB-231 xenograft mouse model were performed to better understand the relationship of the absorbed dose vs. cellular effects, and a comparative study of Monte Carlo dosimetry calculations at the cellular scale for both radiation modalities.

## 2. Results and Discussion

Breast cancer is a complex and heterogeneous disease categorized into three major subtypes based on the presence or absence of molecular markers for estrogen (ER), progesterone (PR), or human epidermal growth factor 2 (HER2) receptors. These three subtypes have distinct risk profiles and treatment strategies [58,59]. In this study, two representative human breast cancer cell lines were used: SKBR-3, which lacks ER and overexpresses HER2, and MDA-MB-231, a highly aggressive triple-negative breast cancer (TNBC) cell line that lacks ER, PR, as well as HER2 [60]. Although breast cancer is the most common cancer in women worldwide, there have been few studies investigating the use and feasibility of BNCT and PBFT in this type of cancer. Preliminary studies pointed out that BNCT may be beneficial to treat breast cancer from the early stage to avoid widespread metastatic disease, but further clinical studies are needed [61]. Proton beams represent a potential treatment option for most breast cancer subtypes, including HER2 (+/−) and triple negative. By contrast with photons, a proton beam increases RBE and delivers radiation only to the breast target area, allowing for higher biological effectiveness with reduced damage to surrounding healthy tissues. Ideally, there is no exit dose; therefore, nearby tissues and critical organs like the heart and lungs can be spared [62,63]. Although there is a risk of late cardio-vascular toxicity, the benefit of protons seems to be higher than photons, but more clinical studies are required to confirm the clinical benefit of proton therapy [64,65,66,67,68].

### 2.1. Cytotoxic Activity Evaluation

The cytotoxicity of Na[*o*-FESAN] in breast cancer cells was evaluated to help the selection of the work concentrations for the uptake and irradiation studies. Moreover, the general requirements of therapeutic drugs include, among others, low cytotoxicity and high tumor uptake. The IC_50_ values found after 24 h treatment of the SKBR-3 and the MDA-MB-231 cells with Na[*o*-FESAN] were 83.5 ± 18.6 µM and 103 ± 24.9 µM, respectively.

### 2.2. Cellular Uptake Analysis

The cellular uptake of Na[*o*-FESAN] was measured by ICP-MS in the subcellular cytoplasm and nucleus fractions, after 24 h of exposure to Na[*o*-FESAN] at 50 µM, which allowed for assessing both B and Fe content. The B-to-Fe ratio of around three found in both cellular compartments aligns with the compound’s stoichiometry, reinforcing its structural integrity and highlighting the importance of nuclear localization for the therapeutic efficacy of both therapies, BNCT and PBFT (Table 1). Moreover, our previous stability studies of Na[*o*-FESAN] in cellular medium showed that the compound did not suffer degradation [25].

**Figure 1:**
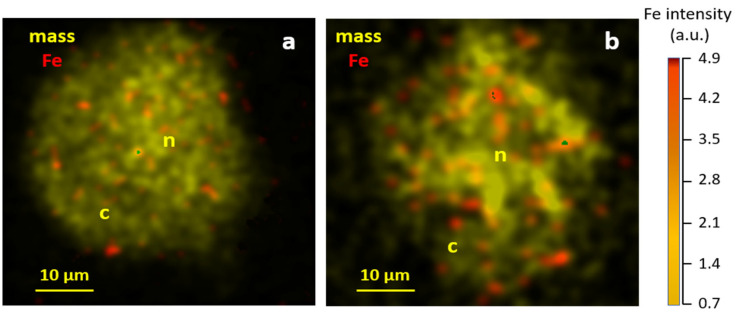
Nuclear microprobe images of Fe distribution (orange for low to red for high intensity) in MDA-MB-231 cells overlaid with mass density (mass): (**a**) Control; (**b**) incubated 24 h with 50 µM Na[*o*-FESAN]. Regions of high-density nucleus (n) and low-density cytoplasm (c) are indicated.

The visualization of Fe distribution in single cells, as a signature of Na[*o*-FESAN] intracellular trafficking, was also studied by nuclear microscopy, [69,70] as illustrated in Figure 1, for MDA-MB-231 cells. The detection of Fe in both the cytoplasm and, notably, the nucleus is consistent with the cellular uptake of Na[*o*-FESAN]. The concentration of Fe in MDA-MD-231 cells treated with Na[*o*-FESAN] increased by 1.7-fold in the cytoplasm and by up to 2.2-fold in the nucleus, as compared to the untreated control counterparts (average Fe concentration in the cytoplasm on a dry weight basis: 250 ± 30 µg/g vs. 150 ± 10 µg/g in controls; in the nucleus: 430 ± 90 µg/g vs. 200 ± 30 µg/g in controls). Therefore, the surrogate Fe map for Na[*o*-FESAN] showed a preferential localization of Fe towards the cell center, suggesting that the nucleus is a cellular target for this compound. This result is relevant for its potential use as a radio enhancers.

The uptake studies in single cells, indicating an increased concentration of Na[*o*-FESAN] in the nucleus, align with the theoretical studies reported by Tagliazucchi M. and Szleifer I. [71], which emphasized the critical role of charge and hydrophobicity in mediating the translocation of model particles through the nuclear pore complex, supporting the observed experimental results reported here. The Fe subcellular distribution showing the nucleus as a cellular target could be related to the interaction between [*o*-FESAN]^−^ and ds-DNA [42,56]. These results confirm the efficacy of carrier-free [*o*-FESAN]^−^ uptake, providing a quantitative approach to the number of compounds internalized in the cells and their subcellular localization. Results are also in line with previous findings that confirm the translocation of [*o*-FESAN]^−^ across planar lipid bilayers in the absence of any receptor and under zero applied voltage [25].

The results presented above align with atomistic MD simulations of [*o*-COSAN]^−^ which show that amphiphilic cobaltabis (dicarbollide) anions can efficiently cross bilayer membranes that are impermeable to ions in a ‘carrier-free’ manner [72,73]. This may be attributed to a cooperative flip-flop mechanism, suggesting a novel ion permeation process that explains both the previous and the present experimental observations [74].

In this study, we have observed that [*o*-FESAN]^−^ crosses the membranes of the studied breast cancer cells that possess the EGFR receptor. This may suggest that EGFR expression in these cells further facilitates the transport of [*o*-FESAN]^−^, like what was reported for the hybrid oligonucleotide (anti-EGFR) conjugate of [*o*-FESAN]^−^ [50].

### 2.3. Biodistribution Studies

Biodistribution studies allowed tracking Na[*o*-FESAN] in an animal model, providing fundamental pre-clinical information on this prospective therapeutic agent. These studies are particularly relevant in the context of BNCT and PBFT, since they provide qualitative and quantitative information about the biodistribution of Na[*o*-FESAN], which is of utmost importance to determine the efficacy of these radiation therapies, defining their localization and accumulation in the target organs and tissues for clinical translation [4].

**Figure 2:**
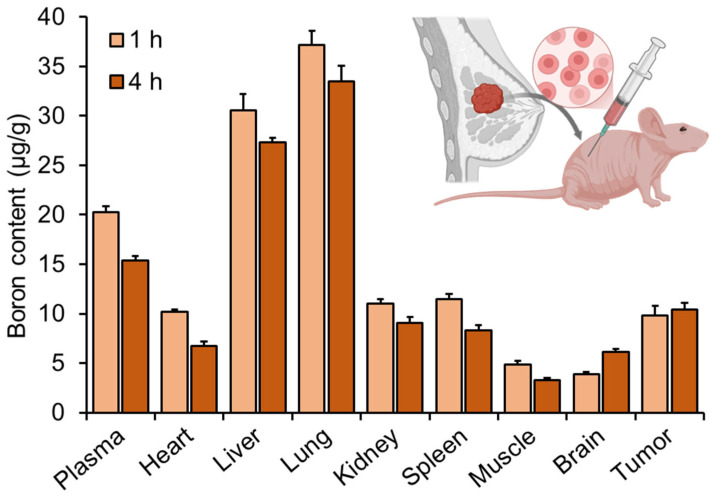
Biodistribution of Na[*o*-FESAN] (7.5 mg B/kg, i.v.) in MDA-MB-231 xenografts (Balb/cSlc-nu/nu, female). Boron concentration is expressed as µg B/g tissue (mean ± SD, n = 6) at 1 and 4 h post-injection.

The biodistribution of Na[*o*-FESAN] (7.5 mg B/kg, i.v.) was evaluated in an MDA-MB-231 breast cancer xenograft model, and the results at 1 and 4 h are shown in Figure 2. After 1 h of treatment, high boron concentrations in each organ were observed in the Na[*o*-FESAN] treated mice that decreased at 4 h post-injection. Although the clearance of Na[*o*-FESAN] was slow in blood and main organs (heart, liver, lung, kidney, spleen, and muscle), the tumor uptake remained relatively high. Notably, tumor retention persisted up to 4 h, whereas a marked decrease was already evident in liver and lung, indicating selective persistence in the tumor relative to normal organs.

### 2.4. Radiation Studies

The outstanding Na[*o*-FESAN] chemical and biological properties, which include good chemical stability, water solubility, low toxicity, and a significant tumor retention (~12%) in a breast cancer xenograft mouse model, prompted us to explore the suitability of this small molecule for multimodal cancer therapy. Taking advantage of their boron-rich content, namely the stable isotopes of natural boron, ^10^B and ^11^B, we performed studies with Na[*o*-FESAN] to evaluate its application in BNCT (^10^B(n,α)^7^Li; Q = 2.31 MeV) and PBFT (^11^B(*p*,α)αα; Q = 8.7 MeV).

#### 2.4.1. Neutron Irradiation—BNCT

BNCT can selectively destroy malignant cells while sparing adjacent normal cells and tissues by delivering a single fraction of high-LET radiation. The success of BNCT relies on two requirements: (i) selective delivery of sufficient amounts of B (^10^B) to the tumor site with negligible amounts to normal tissues; (ii) availability of thermal neutrons with appropriate energy to accomplish the reaction [14]. The ideal boron compound must have high selectivity to tumors and low toxicity to normal tissues, longer retention in tumor cells, and rapid clearance in normal tissues [75]. In silico studies employing Monte Carlo simulations demonstrate that when cells are exposed to neutron reactions, the energy deposition in the cell nucleus leads to a significantly more efficient cell killing effect as compared to a uniform distribution throughout the entire cell [76]. Based on the findings from ICP-MS studies, which revealed accumulation of Fe and B in the cytoplasm and nucleus following the uptake of Na[*o*-FESAN] by breast cancer cells, neutron irradiation was conducted to explore and understand the implications of these observations.

**Figure 3:**
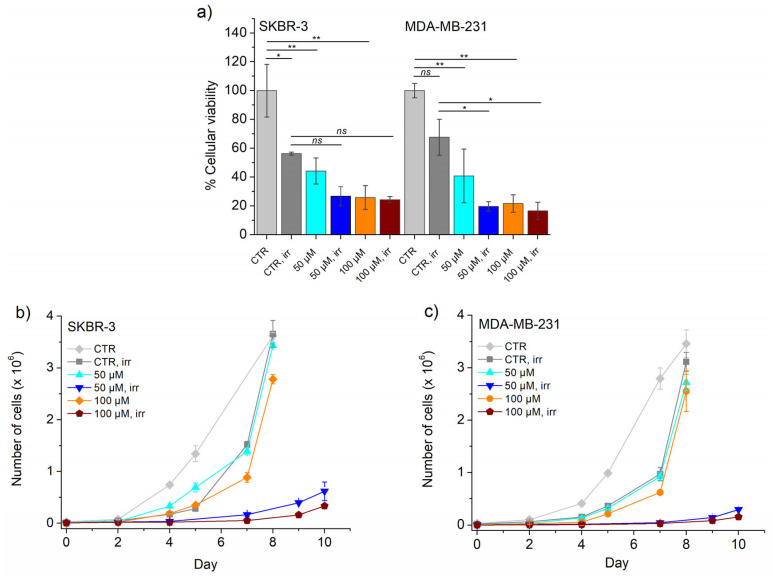
Cellular viability (**a**) and survival (**b**,**c**) of untreated (CTR) and treated with Na[*o*-FESAN] at 50 and 100 µM for 24 h, followed or not by irradiation with thermal neutrons for 15 min at 30 kW reactor power. Results are mean ± SD of at least two experiments with six replicates per condition. Statistical analysis was performed by using Student *t*-test: not significant (ns), *p* > 0.05, * *p* ≤ 0.05, ** *p* ≤ 0.01.

For neutron irradiation, SKBR-3 and MDA-MB-231 cells were incubated with Na[*o*-FESAN] at 50 and 100 µM and were irradiated with thermal neutrons at the Triga Mark II nuclear reactor at the University of Pavia. Figure 3 shows the percentage of cells that are viable at 24 h post neutron irradiation, with respect to the non-irradiated control cells (CTR, 50 µM and 100 µM Na[*o*-FESAN] groups). Only for MDA-MB-231 cells, the number of viable cells after Na[o-FESAN] treatment and irradiation was significantly different with respect to the non-treated cells after irradiation. Instead, cells incubated for 24 h with 100 µM Na[*o*-FESAN] alone showed a high cytotoxicity that did not increase after irradiation. However, although the surviving cell numbers were similar, the proliferation rate of cells that survived irradiation showed an interesting behavior. Both surviving SKBR-3 and MDA-MB-231 cells that were treated with Na[*o*-FESAN] (at both 50 and 100 µM) and then irradiated with neutrons showed a marked inhibition of proliferation (Figure 3b,c). This clearly indicates the important contribution of neutron capture to induce a long-term cytotoxic effect. Another important point is that the toxic effect was observed using Na[*o*-FESAN] containing natural B. In fact, the amount of internalized B measured by ICP-MS after 24 h incubation with 50 or 100 µM Na[*o*-FESAN] contained only 19.9% of the active ^10^B isotope. Assuming that in the case of epithelial tumors (with a diameter ranging from 15–20 µm), a density of ca. 10^8^ cells for cm^3^ is a reasonable number, [76] from the data obtained by ICP-MS analysis, it was possible to calculate the amount of ^10^B internalized in cells expressed in ppm (µg/g). Table 2 shows that these values are not particularly high, and considering that the natural boron contains only 19.9% of ^10^B, they can be further improved using Na[*o*-FESAN] enriched in ^10^B.

Despite the low mean ^10^B concentration (ppm) in both cell lines, a marked inhibition of proliferation was observed after boron neutron irradiation.

The amount of ^10^B internalized from the metallacarborane compound Na[*o*-FESAN] in breast cancer cells (MDA-MB-231 and SKBR-3) is lower than the theoretical thresholds considered optimal (~20–30 µg/g tumor) for BNCT efficacy, as established for other BNCT agents. However, our experimental data clearly demonstrate that even this lower intracellular concentration is sufficient to achieve an efficient BNCT effect. Our explanation for this finding is based on the unique physicochemical and biological properties of the anionic [*o*-FESAN]^−^ cluster, which interacts with double-stranded DNA in an intercalative mode [56].

In the present work, we show that the [*o*-FESAN]^−^ cluster not only can freely cross the cell membranes but also remains localized in the nucleus due to its intercalative binding to DNA. Importantly, theoretical Monte Carlo simulations [77] have reported that BNCT efficacy is significantly enhanced when ^10^B atoms accumulate within the cell nucleus, near DNA, than when the same amount of ^10^B atoms is uniformly distributed in the cell. Therefore, the combination of these two unique properties—nuclear accumulation and DNA intercalation—provides a plausible explanation for why [*o*-FESAN]^−^ exhibits efficient BNCT effects even at intracellular ^10^B concentrations below the conventional threshold (~20–30 µg/g tumor).

To the best of our knowledge, no other BNCT agents tested so far have been shown to exhibit such DNA-interactive behavior. This unique nuclear targeting may underlie the unexpectedly high BNCT efficacy of Na[*o*-FESAN] [56].

#### 2.4.2. Proton Irradiation

**Figure 4:**
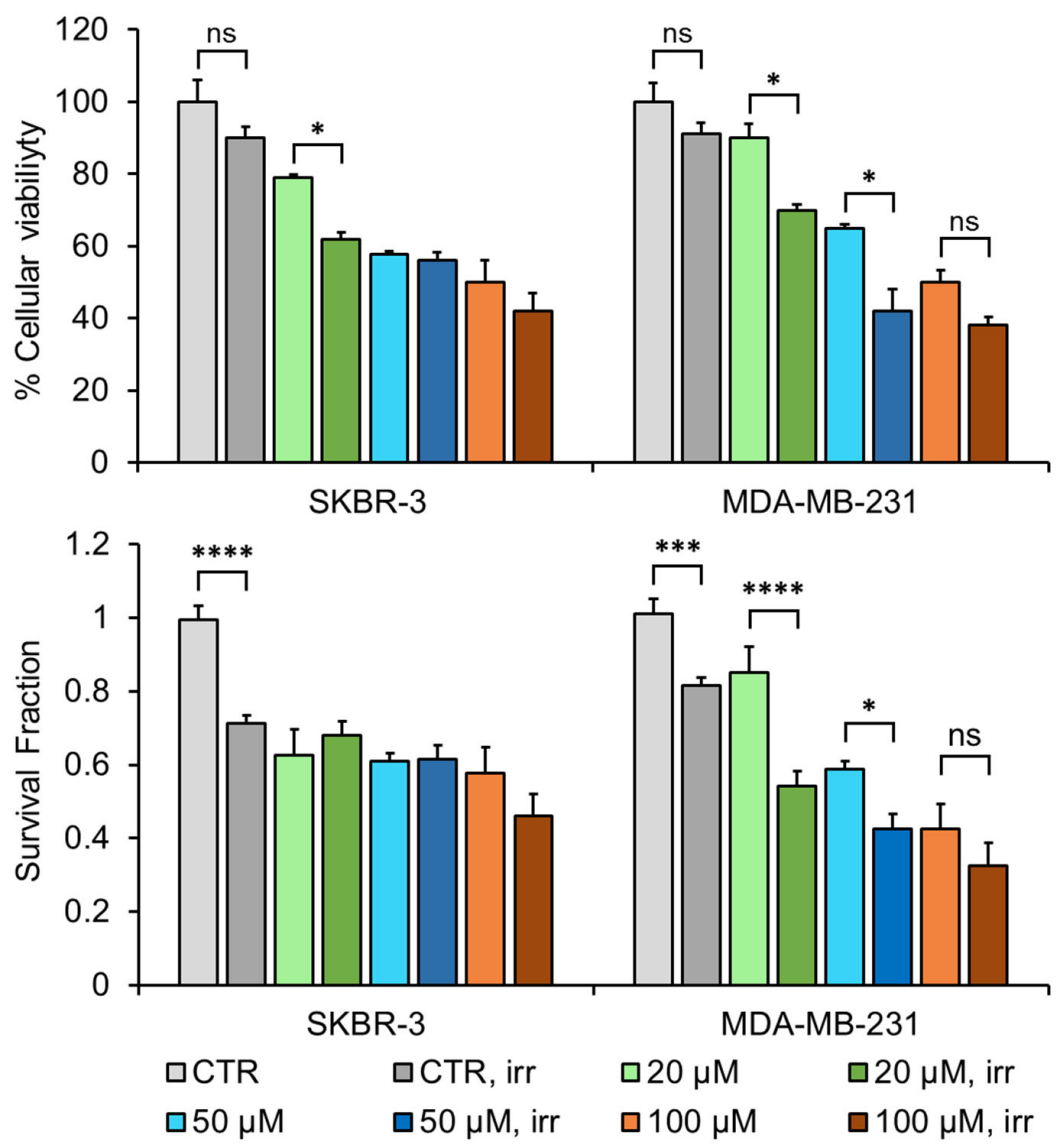
Cellular viability (**upper panel**) and survival (**lower panel**) after treatment with Na[*o*-FESAN] at 20, 50, and 100 µM for 24 h followed by proton irradiation (0.235 Gy). Results are mean ± SD of at least two experiments with six replicates per condition. Statistical analysis was performed by using Student *t*-test: not significant (ns), *p* > 0.05, * *p* ≤ 0.05, *** *p* ≤ 0.001, **** *p* ≤ 0.0001.

The cell-killing efficiency of Na[*o*-FESAN] was evaluated after irradiation of the SKBR-3 and MDA-MB-231 cells with protons (Figure 4). The viability and survival assays were also used as the two most important endpoints of the radiobiological effects [78].

Cells were previously treated for 24 h with Na[*o*-FESAN] at concentrations ranging from 20 µM to 100 µM. As depicted in Figure 4, the cellular viability loss after irradiation is dose-dependent and does not differ much between the two breast cancer cells. For the MDA-MB-231 breast cells at 50 µM, the cellular viability loss after irradiation was ca. 20% relative to its non-irradiated control (Figure 4).

### 2.5. Cellular Effects of Proton Irradiation

Radiation therapy (RT) has multiple effects on cancer cells, both direct and indirect. The direct effects involve DNA, leading to potentially irreparable DNA damage and cell death caused by interaction with radiation. Indirect effects occur when radiation interacts with water molecules—the major constituent of cells and other organic molecules, generating free radicals that further contribute to cellular damage [79].

#### Reactive Oxygen Species (ROS)

Ionizing radiation induces oxidative stress through the radiolysis of water, leading to the generation of reactive oxygen species (ROS). One of the primary mechanisms of radiation therapy is tumor growth inhibition through the action of ROS. When ROS levels (H_2_O_2_, OH^•^, O_2_^•−^) rise to a sufficiently toxic threshold, cellular structures are damaged, triggering cell death pathways such as apoptosis or necrosis. Prolonged oxidative stress following radiation exposure can modify biomolecules such as proteins, lipids, and DNA, disrupting their functions and causing adverse cellular effects.

**Figure 5:**
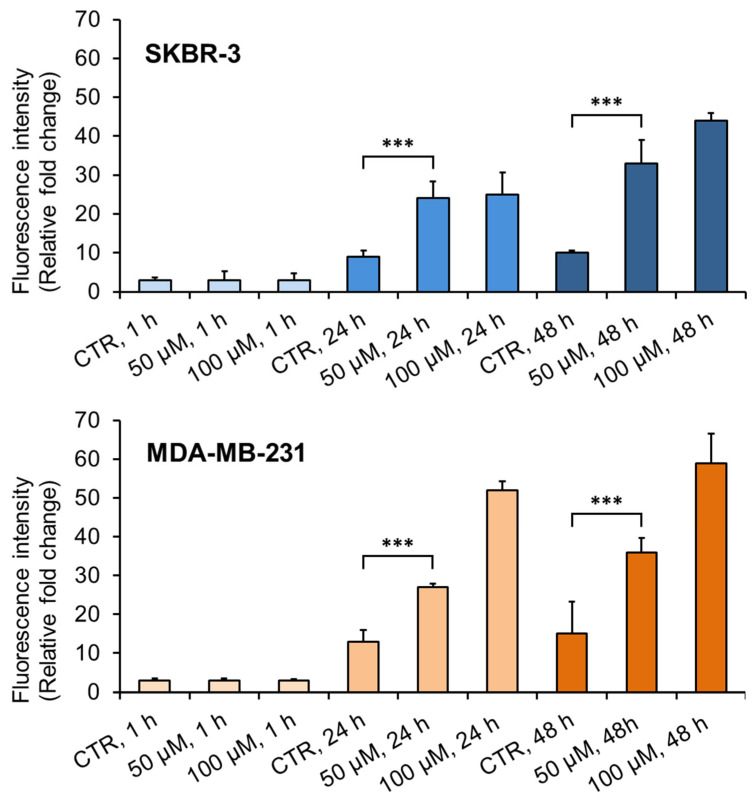
ROS production as fold change relative to controls (non-irradiated cells) measured at 1, 24, and 48 h after proton irradiation (0.235 Gy) in SKBR-3 (**up**) and MDA-MB-231 cells (**down**). Cells were treated with Na[*o*-FESAN] at 50 and 100 µM for 24 h before irradiation. Bars represent the fold change relative to the non-irradiated conditions: cells only in culture medium (controls, CTR) or cells treated with Na[*o*-FESAN] at 50 and 100 µM, and are presented as mean ± SD of two independent assays. Statistical analysis was performed by using Student *t*-test: *** *p* ≤ 0.001.

Our results showed that proton irradiation following incubation with 50 and 100 µM Na[*o*-FESAN] increased ROS levels in a dose-dependent manner compared to the non-irradiated controls over a post-irradiation period (up to 48 h) (Figure 5). In non-irradiated cells (with/without treatment), no appreciable ROS formation was observed. This suggests that the boron compound taken up by the cells has a synergistic effect on ROS generation following proton irradiation with higher-LET radiation at the Bragg peak zone, which could contribute to enhanced cell damage.

### 2.6. TEM Studies

**Figure 6:**
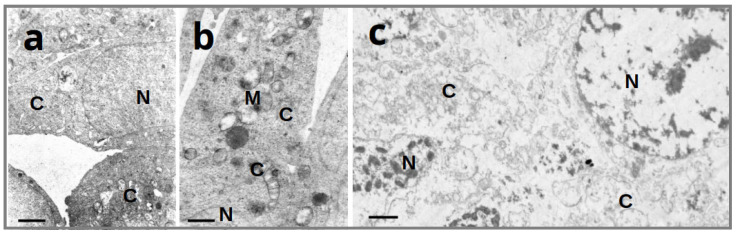
Comparative studies by TEM of the cellular morphological alterations in MDA-MB-231 cells, before (**a**,**b**) and 2 h after irradiation with proton beams (**c**). Control (no treatment) (**a**) showed cells with normal morphology, and Na[*o*-FESAN] treated cells (**b**) showed vesiculation of cellular organelles but no signs of cytotoxicity. Cells treated with Na[*o*-FESAN] and protons (**c**) showed extensive areas of necrosis, including nuclear and cytoplasmic edema and membrane disruption. N—nucleus; M—mitochondria; C—cytoplasm. Bars = 1 µm.

Transmission electron microscopy (TEM) was used to assess the structural integrity of MDA-MB-231 cells and to examine the effects at the organelle level before and 2 h after irradiation with proton beams (2.0 MeV). As shown in Figure 6, Na[*o*-FESAN] treated cells maintain a viable structure with a moderate amount of vacuolization often resulting from mitochondrial damage (Figure 6b), and when irradiated with protons, the cells enter necrosis with marked changes to all cellular components and loss of osmotic control (Figure 6c).

### 2.7. Dosimetry Studies

#### 2.7.1. Proton Dosimetry

**Figure 7:**
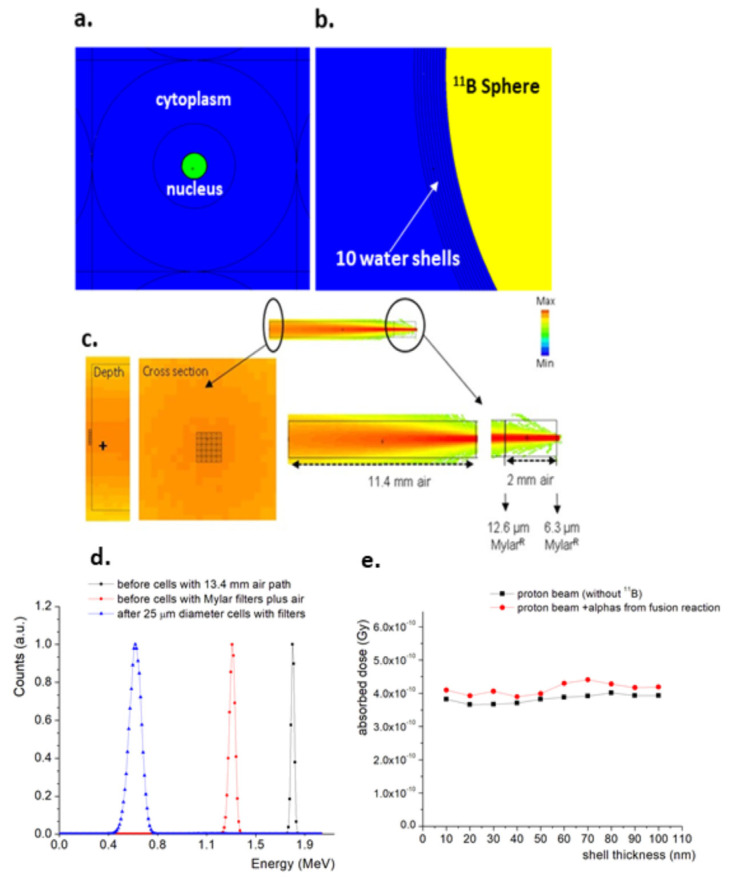
Monte Carlo (MC) simulation and dosimetry calculations for proton irradiation. (**a**) Schematic representation of the MCNP6.1 design of the cytoplasm (cy), nucleus (nu) and ^11^B sphere (green circle) inside the nucleus; (**b**) detail of the MCNP6.1 design of the 10 water nano-shells, used for dose enhancement calculation around a ^11^B sphere; (**c**) MCNP6.1 MC simulations showing the beam cross section, divergence and depth (cross) for the proton irradiation setup (with air path plus filters and cell layer) tuned to reach the ^11^B(*p*,α)αα nuclear reaction near Ep = 0.675 MeV at the sample cell layer; (**d**) Proton energy spectra considering different filters (according to the setup represented in (**c**)) and cell layer (cell diameter of 25 µm water-equivalent). The proton–boron resonance energy range is highlighted in red in the energy axis; (**e**) absorbed dose in consecutive 10 nm water shells for protons, in the absence and presence of boron.

The aim of the dosimetry study was two-fold: (1) To provide an estimation of the cellular dose, since it could greatly affect the absorbed dose vs. cellular effects-relationship and consequently also the models of DNA repair and mis-repair mechanisms, cell killing effects, among others [80]; (2) to estimate the radio enhancement effect (absorbed dose enhancement) that ^11^B could have on the irradiation of tumor cells with protons. For MC simulations, we considered a proton beam of 2 MeV energy (see MC simulation setup in Figure 7c), with a proton flux of 2E7 protons/s (irradiation time of 10 s). With these irradiation parameters, an average absorbed dose of about 0.235 Gy, both in the nucleus and in cytoplasm, was calculated.

Regarding the cell absorbed dose estimations, the calculated values seem to correlate in a reasonable way with the cell studies reported in Figure 4 (viability and survival results have the same trend). Specifically, the viability percentage differences between the Control (CTR) and CTR irradiated are small (about a 10% decrease), reflecting the relatively low absorbed dose delivered with proton irradiation. However, when considering the different concentrations of [*o*-FESAN]^−^, the radiosensitizer effect is evident, confirming the presence of other mechanisms that should be studied to better understand the [*o*-FESAN]^−^ radiosensitization effect.

Relatively to the radiation dose enhancement study, the increase in dose in 10 nanometric water shells around the radio enhancer material (Figure 7b) was assessed through MC simulations. The presence of ^11^B inside the cell triggers the α-particles production, the cascade of low-energy electrons and secondary particles emitted in proximity of the radio-enhancing material [81,82].

However, MC simulations show that the effect of the proton-^11^B fusion reaction seems to be negligible (Figure 7e), probably due to the fact that the proton flux, together with the ^11^B amount, is not effective enough to produce a sufficiently high α-particle flux, capable of causing cell damage [82,83]. Nevertheless, it is safe to say that there are many limitations in radiation dosimetry, both for experimental and theoretical calculations. Experimental results can include up to 30% uncertainties, among others, due to repeatability and geometry setup [80]. MC simulations could present some uncertainties that are linked more to the lack of cross section data for the specific physics events considered in this study (e.g., production of low-energy electrons and production of α-particles in the proton-^11^B fusion reaction). It is essential to include in MC simulations reasonably accurate cross section reactions for boron-enhanced proton therapies. This may not always be the case, particularly for low-energy reactions. Physics lists commonly used often rely on hadronic models suitable only above hundreds of MeV proton energy. Namely, cross section at low energy is either missing or highly unreliable [82,83].

#### 2.7.2. Neutron Dosimetry

For neutron dosimetry, the mixed (n + γ) radiation field inside the LENA-modified thermal column has been widely and carefully measured and characterized as reported in Bortolussi S. et al. [77]. During cell irradiations, detectors to monitor “in real time” the neutron flux at the irradiation position were not inserted, mainly to avoid further perturbation of the neutron field due to the presence of neutron highly sensitive detectors/materials. Therefore, these measurements have been validated with the MC model of the reactor, routinely used to estimate the doses delivered to cell samples.

The absolute absorbed doses (Gy) and percentage fraction (%) are reported in column headers # 2, 4, 6, and 8 of Table 3, for each measured ppm. In terms of absolute dose values, the dose components due to endogenous elements of the biological samples (N14 and H1) and the background gamma contamination due to the thermal column structure are unchanged for the different ^10^B concentrations. On the contrary and as expected, if we consider the absolute values, as well as the respective percentages, the dose values increased as ^10^B increased, with increasing [*o*-FESAN]^−^ concentration. Due to the very small sample irradiated, the dose contribution coming from the 478 keV photons emitted after 94% of ^10^B capture reactions is always negligible due to the non-equilibrium conditions in which these photons are emitted and the very thin layer of material in which the energy should be deposited.

## 3. Materials and Methods

### 3.1. Chemicals

The sodium salt of ferrabisdicarbollide Na[*o*-FESAN] was synthesized from [NMe_4_][*o*-FESAN] by using cationic exchanging resin as reported [84]. The synthesis of [NMe_4_][*o*-FESAN] was achieved by the complexation reaction of the dicarbollide ligand ([7,8-C_2_B_9_H_11_]^2−^) with FeCl_2_ as reported in the literature [85]. Other chemicals used were purchased from Merck (Darmstadt, Germany).

### 3.2. Cell Lines, Cell Culture Conditions, and Na[o-FESAN] Stock Solutions

The human breast adenocarcinoma cells SKBR-3 (HER^2+^) and MDA-MB-231 (triple negative, ER^−^, PR^−^, HER^2−^) were obtained from ATCC(American Type Culture Collection, Manassas, VA, USA). For the biological experiments, cells were cultured in Dulbecco’s Modified Eagle’s Medium (DMEM+ GlutaMAX™ high glucose) (Thermo Fisher Scientific, Inc., Waltham, MA, USA) supplemented with 10% fetal bovine serum (FBS) and maintained at 37 °C in a 5% CO_2_ humidified atmosphere. Fresh stock Na[*o*-FESAN] solutions (10 mM) were prepared in DMEM media without FBS. Serial dilutions from the stock were prepared in medium supplemented with 10% FBS.

### 3.3. Cellular Viability Before Irradiation (Concentration Range Selection)

Before the irradiation studies, the concentrations of Na[*o*-FESAN] were selected. SKBR-3 and MDA-MB-231 cells were counted with a Neubauer chamber using Trypan Blue exclusion and a Zeiss Primovert microscope, seeded in 96-well plates (1–2 × 10^4^ cells/200 µL medium) and incubated at 37 °C for 24 h to adhere. Then, the medium was discarded, and cells were incubated with Na[*o*-FESAN] at serial concentrations in complete medium in the range 1–300 µM for 24 h. After incubation, the cellular viability was determined using the MTT assay as previously described [25].

### 3.4. Cellular Uptake

#### 3.4.1. Uptake by ICP-MS

SKBR-3 and MDA-MB-231 cells (~10^6^ cells/2 mL) were incubated with Na[*o*-FESAN] 50 µM for 24 h in 6-well plates. The cellular fractions, nucleus and cytosol, were obtained using Igepal^®^ ca-630 (Sigma-Aldrich, Darmstadt, Germany). Briefly, after treatment, cells were washed with PBS and collected by centrifugation. Then, the cells were lysed with buffer containing 50 mM Tris (pH 7.4), 250 mM NaCl, 5 mM EDTA, 50 mM NaF, 1 mM Na_3_VO_4_, 0.02% NaN3 and 1% Igepal for 30 min. on ice, with constant vortexing for 10 min. After centrifugation at 400× *g* for 10 min, 4 °C, the supernatant (cytosol) and the pellet (nucleus) fractions were obtained. The levels of B and Fe in each fraction were measured by a Thermo X-Series Quadrupole ICP-MS (Thermo Scientific, Birmingham, UK) and were expressed as ng of B or Fe/10^6^ cells (Table 1).

#### 3.4.2. Single Cell Uptake by Micro-PIXE

The distribution of Fe in single MDA-MB-231 cells (controls and after 24 h incubation with 50 µM Na[*o*-FESAN]) was assessed by nuclear microscopy imaging performed at the nuclear microprobe setup of the CTN/IST Van de Graaff accelerator [69,70]. In brief, cells were seeded on silicon nitride membranes (Silson Ltd., Southam, UK). Images of elemental distributions (such as Fe) and morphology (mass density) were created using different techniques simultaneously by scanning a 2.0 MeV proton beam focused to micrometer dimensions over the sample surface. This enables the colocalization of cell features and Fe quantification by PIXE, which is useful for assessing cellular uptake and elemental compartmentalization. The data acquisition, including imaging processing and spectral analysis, was performed using DAQ-3 software (version 3) (Oxford Microbeams Ltd., Bicester, UK).

### 3.5. Biodistribution of Na[o-FESAN] in MDA-MB-231 Xenograft Models

All animal experiments were approved by the Animal Care and Use Committee of the Institute of Science Tokyo and performed in accordance with the Guidelines for the Care and Use of Laboratory Animals as stated by the Institute of Science Tokyo (Approval number “D2021007”). All animals were maintained under controlled conditions of temperature and humidity with ad libitum water and feeding. MDA-MB-231 tumor-bearing mice (Balb/cSlc-nu/nu, female, 5–6 weeks old, 14–20 g, Sankyo Labo Service Co., Ltd., Hamamatsu, Japan) were prepared by injecting subcutaneously (s.c.) a suspension of MDA-MB-231 cells (8.0 × 10^5^ cells/mouse) directly into the right thigh. The mice were kept on a regular chow diet and water, and maintained under a 12 h light/dark cycle in an ambient atmosphere. When the tumor diameter became 5 to 7 mm, the mice were injected via the tail vein with 200 µL PBS solution of Na[*o*-FESAN] (7.5 mg B/kg). At 1 and 4 h after injection, the mice were lightly anesthetized, and blood samples were collected by cardiac puncture. The mice were then sacrificed by cervical dislocation and dissected. Heart, liver, lung, kidney, spleen, muscle, brain, and tumor were excised, washed with 0.9% NaCl solution, and weighed. The excised organs were digested with 1 mL of conc. HNO_3_ at 90 °C for 1 h, and then the digested samples were diluted with distilled water. After filtering through a hydrophobic filter, boron concentration was measured by inductively coupled plasma optical emission spectroscopy (ICP-OES) using an iCAP 7400 Duo elemental analyzer (Thermo Fisher Scientific Inc., Waltham, MA, USA).

### 3.6. Neutron Irradiation Experiments

SKBR-3 and MDA-MB-231 cells were seeded in 25 cm^2^ flasks (12 flasks for each cell line) at a density of 6.5 × 10^5^ cells. After 24 h from seeding, 4 flasks of each cell line were incubated in the presence of Na[*o*-FESAN] for 24 h at three different conditions: (1) with medium only (CTR cells); (2) with 50 µM Na[*o*-FESAN]; (3) with 100 µM Na[*o*-FESAN]. Cells were then washed with PBS, and a new medium was added. At that point, cells were irradiated at the thermal column for 15 min with a reactor power of 30 kW and compared with cells incubated under the same conditions but non-irradiated. The thermal column of the TRIGA Mark II reactor at the University of Pavia (Italy) was used for neutron irradiation of cells. Two flasks containing treated and two flasks containing non-treated control SKBR-3 and MDA-MB-231 cells were used for this experiment. At the end of the irradiation, the medium was discarded, replaced with a fresh one, and all the flasks were placed at 37 °C in a humidified atmosphere of 5% CO_2_.

### 3.7. Cellular Viability and Survival Assays After Neutron Irradiation

Twenty-four hours after irradiation, cells were detached with 0.05% trypsin and 0.02% EDTA, and cell viability was evaluated using the trypan blue exclusion test and reported as the percentage of cells observed in treated and/or irradiated samples with respect to that observed in control cells. Then, around 3 × 10^4^ SKBR-3 and MDA-MB-231 cells from each differently treated flask were seeded in 6 cm diameter culture dishes. The cell growth was followed for 10 days, and at pre-determined times, the cells were washed with PBS, detached with 0.05% trypsin and 0.02% EDTA, and transferred into falcon tubes. Then, cells were sonicated for 30 s at 30% power in ice, and the total cell protein concentration (that is, proportional to the number of cells) from cell lysates was determined by the Bradford method, using bovine serum albumin as a standard. The cell growth rate was calculated as follows: [(number of cells(t_i_) − number of cells(t_0_))/number of cells(t_0_)] × 100 where (t_i_) is the number of cells measured at the various time intervals and (t_0_) is the number of cells at the starting point (t = 0) of the proliferation assay.

### 3.8. Proton Irradiation Experiments

SKBR-3 and MDA-MB-231 cells (1–2 × 10^4^ cells/well) were seeded into 96-well plates as described above, ensuring uniform cell distribution in a monolayer and incubated with Na[*o*-FESAN] at selected concentrations for 24 h before irradiation with protons. Proton irradiation was performed in air using the external microbeam facility at the CTN/IST Van de Graaff accelerator. Briefly, a 2.0 MeV proton beam focused to ~70 × 70 µm^2^ was raster scanned over the cell monolayer after partially removing culture medium, as described elsewhere [25,69,70].

The proton beam energy was tuned to 675 keV (main resonance energy of the proton–boron nuclear reaction) within the cell layer, controlling air path length and using Mylar^®^foils (Tekra, LLC, New Berlin, WI, USA) as attenuators. For the set experimental conditions, an average LET of 26.4 ± 0.9 keV/µm and an estimated dose of 0.235 ± 0.013 Gy (MC dosimetry study) were delivered to irradiate cells in each well.

### 3.9. Cellular Viability and Survival Assays After Irradiation with Protons

The viability assay was performed directly in the 96-well plates 72 h after irradiation, using the MTT assay [25]. The colony formation assay or cell survival assay was performed immediately after irradiation. After irradiation, cells were trypsinized from the 96-well plates and the number of cells counted. Cell suspensions were then seeded in 6-well plates (3 wells per condition). The number of cells was in the range 100–200, dependent on the concentration used, i.e., 100 for control (no treatment) and the lowest and intermediate Na[*o*-FESAN] concentrations, and 200 for the highest. After 11 days of incubation at 37 °C, cells were treated as previously described [25].

### 3.10. Intracellular ROS

Intracellular ROS levels were measured using the 2′,7′-dichlorodihydrofluorescein diacetate (H_2_DCF-DA) fluorescent probe as previously described [86]. Briefly, cells (~10^4^ cells/well) in 96-well plates (four wells per condition) were treated with Na[*o*-FESAN] for 24 h at 50 µM with or without subsequent irradiation. Then, the probe (10 µM) in colorless DMEM medium (FluoroBrite™ DMEM, Gibco^®^, Waltham, MA, USA) was incubated with the cells for an additional 30 min. at 37 °C. Later, the medium was removed and replaced by a fresh one. The fluorescence intensity was continuously measured up to 48 h with a Varioskan LUX scanning multimode reader (Thermo Fisher Scientific, Waltham, MA, USA). Results of fluorescence (mean ± SD) were expressed as the fold change in fluorescence levels compared with controls (non-irradiated treated cells).

### 3.11. Morphological Analysis by TEM

MDA-MB-231 cells were seeded into 6-well plates at approx. 70% confluence. After 24 h, cells were treated with Na[*o*-FESAN] at 50 µM for 24 h. After incubation, cells were irradiated with a proton beam as described above and processed for TEM analysis 2 h later, following a previously described method [87]. Briefly, the preparation of the cells included several steps of fixation: primary fixation with 3% glutaraldehyde in 0.1 M sodium cacodylate buffer, pH 7.3; secondary fixation with 1% osmium tetroxide in 0.1 M sodium cacodylate buffer, pH 7.3 for 3 h; tertiary fixation and contrasting with 0.5% uranyl acetate in the same buffer for 1 h, dehydration with ethanol and resin (Epon-Araldite) (Merck, Darmstadt, Germany) infiltration and embedding. The stained cells were then photographed and studied using a JEOL 100-SX electron microscope JEOL, Tokyo, Japan.

### 3.12. Proton Simulation and Dosimetric Calculations

Comparative dosimetry studies were performed for BNCT and proton irradiation setups. BNCT dosimetry calculations are described elsewhere [88]. For proton calculations of the absorbed doses, the state-of-the-art MCNP6.1 Monte Carlo (MC) code was used [88,89]. MCNP is the worldwide MC code used and validated for several radiation applications, including external and internal radiation therapy and diagnostic. For this specific task, two types of results were obtained: (i) The absorbed dose calculated at cellular level, for the cell design a standard MIRD [90] configuration was considered, namely a cell composed by cytoplasm (radius of 12.5 µm) and a spherical nucleus (radius of 5 µm), and (ii) the absorbed dose calculated in 10 nanometric water shells (10 nm thickness) in order to study the possible dose enhancement when the cell is irradiated by several radiation qualities and in the presence of radiosensitizers [91] (see Figure 6a,b).

All the dose calculations were performed for a single cell configuration since the knowledge of the absorbed dose for each cell is a more relevant parameter with respect to the average dose calculated in the entire tumor volume. It is well known that tumor heterogeneity could hide the real absorbed dose for each cell, leading to bias in the cell survival vs. absorbed dose curve response [92]. Also, while it is common to report average boron concentrations in the tumor and critical structures, the micro-dosimetric impact of this high-LET radiation therapy is quite dependent on how uniformly the ^10^B is distributed within the tumor. For example, to date, BNCT clinical trials have not focused on evaluating and standardizing the optimal boron dosing delivery regimen. This issue resulted in variations in the clinical results obtained in these trials [32,92].

In all simulations, the number of histories was set in order to have a statistical uncertainty less than 1% for nucleus and cytoplasm dose estimations, and less than 10% for the nanodosimetry results. The uncertainty values were chosen to have a good compromise between computational time and accuracy. The main parameters and design used for the the irradiation setup is reported below.

#### Proton Setup

The MCNP irradiation proton setup is shown in Figure 7c. In this case, the MC model was developed considering the experimental setup previously described, with all the necessary air and Mylar filters, permitting approximately an average proton spectrum energy of about 675 keV (resonance region) inside the cell. To check effectively that the resonance energy region was approximately inside the cells, the proton energy spectrum was calculated at different points, before and after the cells. Figure 7d shows that at the end of the cell diameter, the proton energy spectrum has an average energy of about 600 keV.

To consider the effect of the possible dose enhancement generated by the p + ^11^B → 3α reaction, an ^11^B sphere (radius of 1.5 µm) was placed in the center of the nucleus. Then, 10 water shells were designed around a radiosensitizer material to study the dose variation at the nanometric scale. It is important to stress that the study of energy deposition at the nanometric scale around the radiosensitizer material is a common practice in MC simulation studies since it allows for estimating the dose enhancement (and consequently the increase in possible DNA damage) close to a single radiosensitizer material (in this case ^11^B) [81,91,92,93]. However, it is also important to remark that, to the best of our knowledge, there are no cross section data for the p + ^11^B → 3α reaction in the energy range considered in this study (in the resonant region) suitable for the MC code that we used. To bypass this situation, a two-step MC simulation procedure was adopted. Firstly, the proton beam was simulated, and the energy deposition in the shells was registered. Then, the ^11^B sphere was considered as an α emitter with an average energy of 3 MeV and emitting as an isotropic source. In this α-particle simulation, it is necessary to know the number of α particles that are emitted per incident proton. The yield of the p + ^11^B → 3α reaction was calculated through the following equation [82]:

(1) $$Y_{3\alpha }=\sigma_{R}\times n_{B}$$

where σ_R_ is the reaction cross section in barn and n_B_ is the areal density of ^11^B in Atom/Barn. Considering an interaction material of water thickness of 25 µm and considering an experimental proton flux of 2 × 10^8^, about 18 α-particles are generated.

## 4. Conclusions

Currently, treating breast cancer involves a multidisciplinary approach, often combining surgery, radiation therapy, chemotherapy, hormone therapy, targeted therapy, and immunotherapy. The treatment plan depends on the tumor type, stage, and characteristics, as well as the patient’s overall health and preferences. Radiation therapy may be used at almost every stage of breast cancer and constitutes an effective way to reduce the risk of recurrence. Therefore, the use of new radiation techniques that minimize the doses in surrounding normal tissues and reduce the side effects is a priority. A promising advancement allows for neutron and proton radiation therapies (BNCT/PBFT) to be used after surgical removal of the tumor. By delivering boronated compounds to the breast, followed by radiation, such new procedures are expected to enhance the speed and efficiency of breast cancer treatment.

The development of small molecules with multiple therapeutic applications and improved safety is an urgent need and a challenge in cancer therapy research. On such accounts, we investigate the water-soluble ferrabis(dicarbollide), [3,3′-Fe(1,2-C_2_B_9_H_11_)_2_]^−^ (Na[*o*-FESAN]), a metallacarborane, for multimodal (BNCT/PBFT) cancer therapy. The mechanisms of cell death after irradiation and dosimetry calculations were proposed.

Although Na[*o*-FESAN] internalizes lower amounts of ^10^B than the conventional BNCT threshold, it still produces strong therapeutic effects. This is explained by its ability to cross freely membranes, accumulate in the nucleus, and intercalate into DNA, where ^10^B is most effective. These unique properties account for its surprisingly high BNCT efficiency and distinguish Na[*o*-FESAN] from other boron delivery agents.

To the best of our knowledge, no other BNCT agents tested so far have been shown to exhibit such DNA-interactive behavior. This unique nuclear targeting may explain the unexpectedly high BNCT efficacy of Na[*o*-FESAN].

Our results also evidence the importance of the intranuclear localization of Na[*o*-FESAN] and its DNA binding, which increases the probability of a direct or indirect ionization of nucleotides because of the neutron capture, protons, and other ionizing radiation.

Importantly, our biodistribution studies reveal both opportunities and challenges. While Na[*o*-FESAN] showed clear tumor uptake and retention, its accumulation in the liver, lung, and plasma may raise toxicity concerns. As BNCT and PBFT are typically combined with localized irradiation, such risks could be minimized. However, more extensive pharmacokinetic analyses, in vivo efficacy studies, and future investigations using ^10^B-enriched Na[*o*-FESAN] or the availability of improved derivatives, will be critical to fully establish its therapeutic potential.

For neutron irradiation, the absorbed dose and cellular effects enhancement were evident. For proton irradiation, the absorbed dose enhancement, in line with literature data, is not evident, partially due to the low cross section of the PB reaction in producing an α particle. However, our cellular viability experiments confirm the enhancement effect, showing decreased cell survival. Importantly, increased ROS production and profound cellular structural changes that follow proton irradiation suggest that cell death may be mediated by ROS. Consequently, computational absorbed dose calculations and cellular outcomes upon irradiation support the view of synergistic effects occurring in cells, other than those directly exposed to radiation.

Overall, our encouraging results lead us to anticipate that Na[*o*-FESAN] or novel boron-based small molecule drugs for cancer treatment, with similar DNA binding characteristics, offer unique possibilities to improve and optimize radiotherapy in a multimodal perspective. Therefore, it is expected that these radiation modalities will gain significant development, soon providing healthcare professionals with stronger and well-suited tools for the treatment of breast cancer.

## Figures and Tables

**Table 1:** Cellular uptake (in terms of B/Fe ratio) in the nucleus and cytoplasm fractions of SKBR-3 and MDA-MB-231 cells measured by ICP-MS. The uptake values of B and Fe were expressed in ng/10^6^ cells.

SKBR-3		MDA-MB-231	
B/Fe		B/Fe	
Nucleus	Cytoplasm	Nucleus	Cytoplasm
2.9	2.8	2.7	2.8

**Table 2:** The amount of internalized B measured by ICP-MS after 24 h incubation with 50 or 100 µM of Na[*o*-FESAN], where the ^10^B isotope is 19.9% of total B.

Cell Line	Na[*o*-FESAN] (µM)	B ng/10^6^ Cells	^10^B ppm
MDA-MB-231	50	310 ± 87	6.2 ± 1.7
MDA-MB-231	100	406 ± 113	8.1 ± 2.2
SKBR-3	50	303 ± 73	6.0 ± 1.3
SKBR-3	100	475 ± 114	9.4 ± 2.2

**Table 3:** Neutron dosimetry report for each ppm measured.

Radiation Contribution	Dose (Gy) 6.0 ppm	% Dose	Dose (Gy) 6.2 ppm	% Dose	Dose (Gy) 8.1 ppm	% Dose	Dose (Gy) 9.5 ppm	% Dose
^10^B (94%)	0.865	54	0.894	54	1.17	60	1.37	64
^10^B (6%)	0.066	4	0.068	4	0.089	5	0.105	5
^10^B Tot	0.931	58	0.962	59	1.259	65	1.475	68
^14^N	0.323	20	0.323	20	0.323	17	0.323	15
H scatt	0.081	5	0.081	5	0.081	4	0.081	4
Total *p*	0.404	25	0.404	25	0.404	21	0.404	19
2.2 MeV γ	0.163	10	0.163	10	0.163	8	0.163	8
478 keV γ	0	0	0	0	0	0	0	0
γ background	0.113	7	0.113	7	0.113	6	0.113	5
Total γ	0.276	17	0.276	17	0.276	14	0.276	13
TOTAL	1.611		1.642		1.939		2.155	

## Data Availability

The original contributions presented in this study are included in the article. Further inquiries can be directed to the corresponding authors.
